# Edge Computing for AI-Based Brain MRI Applications: A Critical Evaluation of Real-Time Classification and Segmentation

**DOI:** 10.3390/s24217091

**Published:** 2024-11-04

**Authors:** Khuhed Memon, Norashikin Yahya, Mohd Zuki Yusoff, Rabani Remli, Aida-Widure Mustapha Mohd Mustapha, Hilwati Hashim, Syed Saad Azhar Ali, Shahabuddin Siddiqui

**Affiliations:** 1 Department of Electrical and Electronics Engineering, Universiti Teknologi PETRONAS (UTP), Seri Iskandar 32610, Perak, Malaysia; khuhed_22000210@utp.edu.my (K.M.); mzuki_yusoff@utp.edu.my (M.Z.Y.); 2 Faculty of Medicine, Hospital Canselor Tuanku Muhriz UKM, Cheras 56000, Kuala Lumpur, Malaysia; drrabaniremli@ukm.edu.my (R.R.); aida.widure.mustapha@hctm.ukm.edu.my (A.-W.M.M.M.); 3 Department of Radiology, Faculty of Medicine, Universiti Teknologi MARA, Sungai Buloh 47000, Selangor, Malaysia; hilwa167@uitm.edu.my; 4 Aerospace Engineering Department, Interdisciplinary Research Center for Smart Mobility & Logistics, King Fahd University of Petroleum & Minerals, Dhahran 31261, Saudi Arabia; syed.ali@kfupm.edu.sa; 5 Department of Radiology, Pakistan Institute of Medical Sciences, Islamabad 44000, Pakistan; dr.shahabsiddiqui@gmail.com

**Keywords:** artificial intelligence (AI), Android, computer-aided diagnosis (CAD), cost–benefit analysis, medical imaging, Nvidia Jetson Xavier, real-time deployment, Raspberry Pi, TFLite

## Abstract

Medical imaging plays a pivotal role in diagnostic medicine with technologies like Magnetic Resonance Imagining (MRI), Computed Tomography (CT), Positron Emission Tomography (PET), and ultrasound scans being widely used to assist radiologists and medical experts in reaching concrete diagnosis. Given the recent massive uplift in the storage and processing capabilities of computers, and the publicly available big data, Artificial Intelligence (AI) has also started contributing to improving diagnostic radiology. Edge computing devices and handheld gadgets can serve as useful tools to process medical data in remote areas with limited network and computational resources. In this research, the capabilities of multiple platforms are evaluated for the real-time deployment of diagnostic tools. MRI classification and segmentation applications developed in previous studies are used for testing the performance using different hardware and software configurations. Cost–benefit analysis is carried out using a workstation with a NVIDIA Graphics Processing Unit (GPU), Jetson Xavier NX, Raspberry Pi 4B, and Android phone, using MATLAB, Python, and Android Studio. The mean computational times for the classification app on the PC, Jetson Xavier NX, and Raspberry Pi are 1.2074, 3.7627, and 3.4747 s, respectively. On the low-cost Android phone, this time is observed to be 0.1068 s using the Dynamic Range Quantized TFLite version of the baseline model, with slight degradation in accuracy. For the segmentation app, the times are 1.8241, 5.2641, 6.2162, and 3.2023 s, respectively, when using JPEG inputs. The Jetson Xavier NX and Android phone stand out as the best platforms due to their compact size, fast inference times, and affordability.

## 1. Introduction

The provision of good quality healthcare is a fundamental right of the global population to prevent avoidable loss of life and increase its quality. The experiences from the COVID-19 pandemic demand resilient healthcare systems with the capabilities to not only handle routine care but ensure good quality healthcare even in time of crises and health emergencies [1]. Unfortunately, the healthcare system, especially in developing countries, faces a lot of bottlenecks in terms of the unavailability of infrastructure, resources, and medical experts. Finding solutions to these problems seems to be the most important priority of the hour.

Artificial Intelligence (AI) can significantly assist in this domain [2,3], thereby reducing delays and probability of human errors in diagnosis [2,4]. Medical imaging has revolutionized diagnostic medicine and has now become an indispensable part of diagnosis and treatment process [5]. Research in this domain has significantly matured, and now numerous imaging modalities are available including X-rays, Magnetic Resonance Imaging (MRI), Computed Tomography (CT), Positron Emission Tomography (PET) scans, ultrasounds, etc., to examine anatomical structures. Among the numerous available biomedical imaging modalities, MRI is often considered the most versatile and non-invasive imaging tool for examining the human body. MRI offers high spatial resolution, high soft tissue contrast, tomographic imaging, multidirectional scans, and the integration of anatomical, physiological, metabolic, and functional imaging features. These have assisted neurologists in diagnosing a vast number of disorders [6]. MRI measurements vary widely depending on the technology used, brain scan orientation, and magnetic strength. In addition to planar data that deal with 2D brain images (slices), 3D volumetric MRI data of the entire brain are available for expert and machine examination. These data have been assisting radiologists and medical experts in reaching concrete diagnoses [7].

The very recent surge in big data and data storage and processing capabilities of machines has paved the way for AI to step in for assistance [8]. The massive publicly available annotated data can be used to train Deep Neural Networks (DNNs) to assist radiologists in differential diagnoses, thereby reducing delays in case of pandemics and medical emergencies. Graphics Processing Unit (GPU) technology and parallel processing facilitate the training of such complex networks with humongous data to enable them to produce accurate inferences when tested with fresh data, not seen during training. Once trained adequately, these deep networks can be embedded in Computer-Aided Diagnosis (CAD) tools for real-time use in hospitals. It is crucial to recognize that unlike the old-school approaches, digital diagnostic and intervention techniques have been constantly evolving at an enormous pace. Various concerns have been raised by researchers and end users of mobile medical and health apps from safety, reliability, privacy, efficacy, and regulatory perspectives [9]. The medical and scientific community as well as the governing bodies must ensure that such healthcare applications, with special emphasis on diagnostic apps, are evaluated and regulated to protect end users (patients or healthcare professionals) from any harm or adverse consequences [10]. The next challenge is the selection of an optimal platform to deploy such tools [11].

In case of a lack of personnel and resources, cloud computing can help but introduces latency, i.e., a delay in data transmission and reception over networks. Moreover, data processing over cloud can prove to be expensive in the long run. This can also be rendered unfruitful in the absence of network infrastructure in remote areas. In such cases, edge computing can save the day. The design and development of Single-Board Computers (SBCs) like Raspberry Pi bring the acquisition and processing at the edge of the network in addition to being fairly economical [12]. This offers enhanced security and privacy, as data are processed locally instead of being passed on to remote servers [7]. This can be taken to the next level by using GPU-embedded SBCs, like NVIDIA Jetson Xavier, which have been specially designed to handle AI-based applications. Although these SBCs are powerful, they might not be a suitable choice for training deep learning (DL) models [13]. Nevertheless, some studies have used them successfully for this task [14]. But for obtaining inferences from pretrained models, these devices can prove to be an excellent choice for low-power and secure real-time operations.

Mobile devices/smartphones are another interesting domain for the deployment of such applications, which has not been explored much. They can offer integration of lighter and faster versions of DL models to provide real-time inferences. This can be the most efficient and economical platform since there exists a very wide variety of such gadgets with different specifications and prices, and almost everybody nowadays carries one for everyday use.

From the point of view of AI-based CAD tools, DL architectures can be used to handle two basic problems, namely, the classification and segmentation of medical images [7]. For example, brain MRI images can either be classified as normal or pathological, depending on the number of diseases the classification model is trained with [15]. On the other hand, the contours of brain tumors can be extracted using segmentation to examine their growth in a longitudinal study [16]. Similarly, brain lesions can be segmented in a study aimed at the differential diagnosis of Multiple Sclerosis (MS) and Neuromyelitis Optica (NMO) which produce similar plaques in the brain [17]. The time taken to produce segmentation masks is generally higher than that taken to infer the class of an image [7].

This study aims at investigating the processing time of a brain MRI in both classification and segmentation scenarios in search of an optimal platform for the real-time deployment of CAD tools to assist medical experts in hospitals. Smartphones, which have previously been rarely tested from this perspective, are also included in this study. The performance accuracy of DL models in their baseline forms and the quantized TFLite versions is also compared, along with a cost–benefit analysis of various platforms. Two DL models trained in previous studies are employed for testing. The 12-class classification model embedded in an app called NeuroImaging Sequence Examiner (NISE) [18] identifies the brain MRI sequence and orientation, whereas the segmentation model called NeuroImaging Volumetric Extractor (NIVE) [19,20] is a skull-stripper (brain extractor). The hardware platforms chosen for testing the speed of the models include a gaming laptop Lenovo Legion, NVIDIA Jetson Xavier NX, Raspberry Pi 4B, and an Android smartphone Infinix Note 7. Additional details of the hardware and software used are provided in subsequent sections.

The organization of this paper is such that the prior art in this direction of research is given in Section 2. Section 3 provides details of the hardware, software, DL models, and libraries used along with the adopted methodology. The results and cost–benefit analysis are given in Section 4 and the paper is concluded in Section 5.

## 2. Prior Art

A review of the recent literature suggests that there is ongoing and vigorous research focused on creating CAD tools and implementing them across diverse platforms for practical application. In [13], Civit-Masot et al. discuss a research study that explores the performance of Raspberry Pi 4 and Coral Dev Board in fundus image segmentation. It is suggested that Machine Learning (ML) hardware accelerators can speed up the processing up to 130 times, making them a viable and effective choice for addressing real-time segmentation problems. A more recent work by Isosalo et al. [3] employs NVIDIA Jetson Xavier NX to perform image reconstruction in cone beam CT and cancer detection from mammographic images. Their findings suggest that such operations can be performed by GPU-embedded edge computing devices in reasonable time to assist in diagnostic processes.

Biswas et al. in [21] study the learning and predicting times for COVID-19 detection using Raspberry Pi 4, NVIDIA Jetson Nano and Xavier, and conclude that Xavier is the fastest, as expected, due to its embedded GPU. On a related note, ref. [22] delves into a discussion on various bottlenecks associated with hardware incompatibilities that may impact training speeds on AGX Xavier, Xavier NX, and Nano. Furthermore, Moreira et al. [12] explore the prediction times of multiple Convolutional Neural Networks (CNNs) trained for COVID-19 detection on Raspberry Pi 4.

In [7], the work by Rodrigues Moreira et al. compares the timing and energy performance of Coral Dev Board and NVIDIA Jetson Nano for the segmentation and classification of eye fundus images. The deployment on the devices demonstrates the capability to operate in real-time, achieving segmentation and classification accuracies comparable to high-performance devices like Google Cloud GPUs and Tensor Processing Unit (TPU) but with significantly lower power consumption. The utilization of NVIDIA AGX Xavier for biomedical image applications is highlighted in the referenced work [14,16]. This involves the classification of X-ray images using lightweight neural networks [14]. Additionally, the deployment of NVIDIA AGX Xavier is applied for brain tumor segmentation using CNNs as discussed in the work by Niepceron et al. [16]. It is noted that the NVIDIA AGX Xavier in [14] is employed not only for deployment but also during the training phase of the neural network.

Although the research mentioned thus far approves the performance and use of GPU-embedded edge devices like Xavier for the deployment of AI-based CAD applications, their high cost remains a major issue in their widespread adaptation as an optimal platform. Exploring other options, like smartphones, would therefore be a reasonable idea. Contributing to this exploration, Tobias et al. in [23] present an Android app designed for diagnosing pneumonia. This app employs the MobileNetV2 architecture and takes X-ray images as input for the classification process. Additionally, Chukwu et al. [24] present an Android app development for breast cancer classification using CNNs. These efforts highlight the potential of leveraging smartphones as accessible platforms for AI-based medical applications, thereby addressing cost concerns associated with more specialized hardware. A notable work by Bushra et al. [25] proposes a CNN-based Android app to detect COVID-19 using X-ray images. The goal of such studies is to provide reliable and low-cost CAD tools to assist medical professionals in the diagnosis process. The research work employing Android devices to deploy medical imaging-based CAD tools encourages the idea to include smartphones in the list of embedded systems to be tested in this study for the identification of the optimal platform.

Indeed, there is relatively limited research that offers a direct comparative analysis between SBCs and smartphones. In [26], Cococi et al. undertake a performance comparison of various DNN architectures on both Raspberry Pi and Android devices, utilizing chest X-rays for their evaluation. Similarly, ref. [27] proposes an anamorphic depth embedding-based lightweight CNN for segmenting anomalies in COVID-19 chest CT scans. The authors compare their architecture with other state-of-the-art models and deploy it on diverse platforms, including Raspberry Pi 4, NVIDIA Jetson Xavier, and an Android mobile device, specifically, the Nokia 5.2 plus. While the expected observation is that Xavier performs the fastest, closely followed by the Android device, this research lacks insights into classification tasks and power consumption across the different platforms.

Our research aims to analyze the computational speeds of both classification and segmentation models deployed on a high-end gaming laptop Lenovo Legion, intermediate edge devices NVIDIA Jetson Xavier NX and Raspberry Pi 4B, and a low-end Android device Infinix Note 7. A cost–benefit analysis is also provided along with a comparison of power consumption and flexibility of use. The following section provides details of the materials and methods employed in this research.

## 3. Materials and Methods

This section provides the details of the hardware, software and publicly available datasets employed in this study. Two types of CAD tools, NISE and NIVE, are used for MRI sequence identification and MRI brain extraction, respectively. After training the DL models for both tools, the resulting Directed Acyclic Graph (DAG) network is converted into a Python-importable model for deployment on three platforms: a laptop and two SBCs, specifically, the Jetson Xavier NX and Raspberry Pi. For Android deployment, the model is converted to TFLite format to reduce its size. Two size reduction methods are considered: float-16 model conversion and Dynamic Range Quantization (DRQ). Four performance evaluation parameters, namely, inference time, power consumption, cost, and accuracy, are assessed to determine the optimal platform for real-time deployment.

### 3.1. Devices for CAD Application Deployment

A summary of the devices utilized in this research including the laptop, SBCs, and an Android phone is given in Table 1. The hardware platforms used for comparison in this research include Lenovo Legion Y545 with Intel(R) Core(TM) i7-9750H CPU @ 2.60 GHz processor, 16 GB RAM, and NVIDIA GeForce GTX 1660 Ti GPU.

In addition, we have NVIDIA Jetson Xavier NX 16 GB, with 21 TOPS AI performance, 384-core NVIDIA Volta™ GPU with 48 Tensor Cores, and 6-core NVIDIA Carmel ARM^®^v8.2 64-bit CPU. It has a 16 GB 128-bit LPDDR4x (59.7 GB/s) memory and a 16 GB eMMC 5.1 internal storage. A 128 GB NVME SSD is also used for additional storage. Xavier NX can operate at 10, 15 and 20 W power with 9 modes of operation. The maximum CPU and GPU operational frequencies range from 1200 to 1900 MHz and 510 to 1100 MHz, respectively. In this study, the results for the Xavier platform without GPU refer to operation in the default mode, which is 15 W, 2-core mode. The results with GPU enabled are obtained using the 20 W, 6-core mode.

Raspberry Pi 4B with Broadcom BCM2711, quad-core Cortex-A72 (ARM v8) 64-bit SoC @ 1.5 GHz processor and 8 GB memory is also employed. Additionally, a reasonably priced smartphone—Infinix Note 7—is also used for cost–benefit analysis and comparison. The specifications of this phone include a Mediatek MT6769V/CB Helio G70 (12 nm) Chipset, Octa-core (2x2.0 GHz Cortex-A75 & 6x1.7 GHz Cortex-A55) CPU, Mali-G52 2EEMC2 GPU, and Android 10 Operating System.

### 3.2. Software

Details on the software and its version for the devices used for deployment of the CAD tools are given in Table 2. MATLAB R2022b, Python 3.10.7 and Android Studio Giraffe 2022.3.1 (with Java for coding) are the major software used in this research. MATLAB Graphical User Interface Development Environment (GUIDE) and QT designer with PyQt5 are used for GUI development. The packages used in Python on Lenovo Legion and their respective versions include TensorFlow-2.10.0, TensorFlow-GPU-2.10.0, PyQT5, pydicom, tkinter, nibabel, cv2, among others. NVIDIA CUDA 11.2 and cuDNN 8.1 are used to manage and perform GPU-accelerated operations.

For Xavier NX, Jetpack 5.1.2 is installed using Ubuntu 20.04.6 (Focal Fossa) host machine and NVIDIA SDK Manager 2.0.0, with NVIDIA CUDA 11.4 and cuDNN 8.6. TensorFlow 2.12.0 is used with Python 3.8.10. For Raspberry Pi 4B, python 3.11.2 is used on Debian GNU/Linux 12 (bookworm) with TensorFlow 2.15.0.

### 3.3. CAD Tools: NeuroImaging Sequence Examiner (NISE) and NeuroImaging Volumetric Extractor (NIVE)

An MRI sequence type and orientation identification model trained in a previous study is used in the classification part of this study. MRI data come in a wide variety of contrasts and orientations from hospital sources using different acquisition hardware and protocols. Some file formats (like Dicom) have the capability to store metadata information in the header [28]. These headers contain information like MRI sequence, body part scan, magnet strength, etc., mostly entered manually by technicians at the time of acquisition. This information might not always be accurate, or even be absent altogether in some cases. In addition, to ensure anonymity in research settings, the Dicom tags are removed, rendering important information for data management unavailable [29]. Such additional information and metadata about the MRI scans are not offered by other file formats like NIfTI and Jpeg, which are common file formats used for MRI data sharing. A sequence identification system can be useful in such cases, where a particular sequence might be required for the development of a CAD tool for differential diagnosis [30]. This classification app, called NISE, has the ability to identify T1, T2-weighted, Proton Density (PD) and Fluid-Attenuated Inversion Recovery (FLAIR) sequences in the axial, coronal and sagittal orientations. MobileNetV2 architecture is used in this app for classification with input MR image dimensions of 224 × 224 × 3.

**Figure 1:**
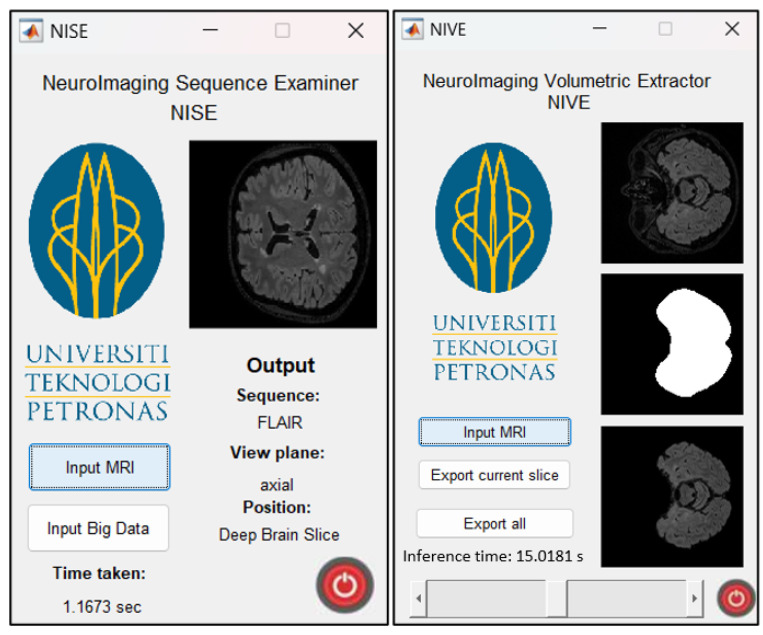
Interface of the NeuroImaging Sequence Examiner (NISE) app (**left**), which displays the sequence, orientation, and relative position of the input brain MRI, alongside the corresponding inference time. The NeuroImaging Volumetric Extractor (NIVE) app (**right**) showcases the input MRI (**top**), the generated brain mask (middle), and the skull-stripped output (**bottom**). The NIVE app also includes a slider for navigating through individual brain slices and an option to save the skull-stripped MRI images.

In addition, a skull-stripping/brain extraction model trained in another previous study, called NIVE, is used for the segmentation part of this study. NIVE accepts raw brain MRI in various contrasts and orientations and removes the extra-cranial tissues including the skull, neck, and orbitals, to extract the brain. This preprocessing of an MRI is vital in various CAD studies, e.g., training DL models for brain lesion segmentation to assist in differential diagnosis of MS and NMO. DeepLabV3+ is embedded in NIVE with input dimensions of 256 × 256 × 3. Both the apps used in this study can accept Jpeg, Png, Bmp and Dicom slices as well as NIfTI whole brain volumes. Both the segmentation and classification TensorFlow models are converted to TFLite versions to embed in the Android app. The MATLAB versions of the two apps are given in Figure 1. The details of the datasets used for the training, validation, and testing of NISE and NIVE, along with those used for time evaluation in this study, are given in Table 3. The test datasets for brain extraction, MRI sequence and orientation identification (classification) apps are performed on MRI scans from SynthStrip dataset [31] and Advanced International Hospital (AIH) dataset [32], in Jpg, Dicom and NIfTI file formats. The performance evaluation on various platforms and processing times are tabulated and analyzed. Other datasets used include NFBS [33], MICCAI 2016 [34], MICCAI 2021 [35], Baghdad [36], IXI [37], and ADNI [38].

### 3.4. Deployment Methods of CAD Tools and Model Reduction

Both the classification and segmentation models are trained using Lenovo Legion in MATLAB. The DAG network in MATLAB is first converted to Python importable model. This is followed by the conversion to TensorFlow Lite model, an optimized FlatBuffer format identified by the ‘.tflite’ file extension, using the TensorFlow Lite converter. This conversion to TFLite for mobile deployment offers certain advantages in terms of reduction in model size and faster performance. This is basically achieved using quantization. The quantized models use lower precision (e.g., 16-bit instead of 32-bit float), leading to benefits during deployment.

**Figure 2:**
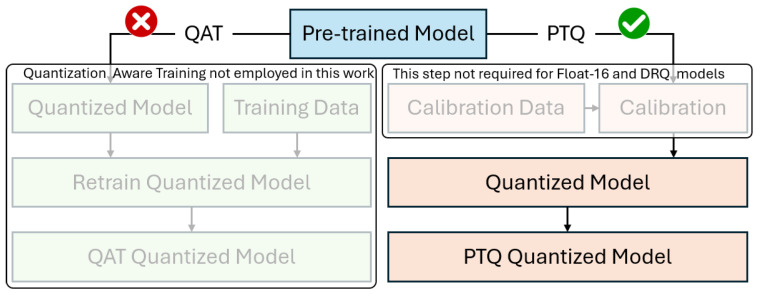
A general comparison between post-training quantization (PTQ) and quantization-aware training (QAT) schemes. The QAT is not employed in this work.

There are two fundamental types of this process, post-training quantization (PTQ) and quantization-aware training (QAT). PTQ is generally preferred due to being easy to use on any trained model, but it has a tendency to degrade model accuracy since the model weights after being quantized (post-training) from 32-bit float to any lower precision (float16 or int8) will surely affect the model output. QAT, on the other hand, is often better for model accuracy but introduces the overhead of modifications in the training process [39]. The general idea of both PTQ and QAT schemes is given in Figure 2. Table 4 presents the most common PTQ options and their benefits. Additionally, Table 4 includes details on QAT, but it is not employed in this work.

Float16 quantization (F16Q) reduces the model size by half by converting weights to 16-bit floating-point values during the TFLite conversion process. This approach allows for a significant reduction in model size with only a slight compromise in accuracy. DRQ further reduces the model size by up to 4× by converting weights to 8-bit precision while keeping activations in the floating-point format. This results in faster inference compared to optimizations that rely solely on floating-point computations. Full Integer Quantization (FIQ), on the other hand, converts 32-bit floating-point values of the weights and activation outputs to the nearest 8-bit fixed-point numbers. This not only produces a smaller model but also increases inference speed, which is particularly beneficial for low-power devices such as microcontrollers. Additionally, this data format is essential for integer-only accelerators like the Edge TPU. However, this strategy tends to have the most significant impact on accuracy.

F16Q and DRQ, being the most gentle in terms of accuracy degradation, are employed in the TFLite conversion process in this research. In F16Q, the weights are quantized from float32 to float16, while in DRQ, they are quantized to int8, resulting in model size reductions of up to 2× and 4×, respectively. Both quantization schemes lead to some reduction in model performance accuracy, with F16Q causing less degradation than DRQ. This performance loss can become more pronounced in smaller networks trained on limited data.

### 3.5. Performance Metric

An accuracy and Dice score comparison between baseline and TFLite models is hence performed in this study for classification and segmentation models, respectively. In the context of image segmentation, the Dice score evaluates the similarity between a predicted segmentation mask and the Ground Truth (GT) segmentation mask. The Dice score ranges from 0, indicating no overlap, to 1, indicating perfect overlap. Mathematically, given two sets, *X* and *Y*, Dice score/Dice similarity coefficient (DSC) can be evaluated as

(1) $$DSC=\frac{2|X\cap Y|}{\left|X|+|Y\right|}$$

where |X| and |Y| are the cardinalities of the two sets (i.e., the number of elements in each set). When applied to Boolean data, using the definition of true positive (TP), false positive (FP), and false negative (FN), it can be written as

(2) $$DSC=\frac{2TP}{2TP+FP+FN}$$

In ML applications, accuracy is a measure of how often a model correctly predicts the outcome. It can be measured on a scale of 0 to 1 or as a percentage. A value closer to 1 indicates decent model performance. In terms of the TP, FP, FN used in (2), accuracy can be expressed as

(3) $$Accuracy=\frac{TP+TN}{TP+TN+FP+FN}$$

where, TN stands for true negative.

### 3.6. Experiment Setup

**Figure 3:**
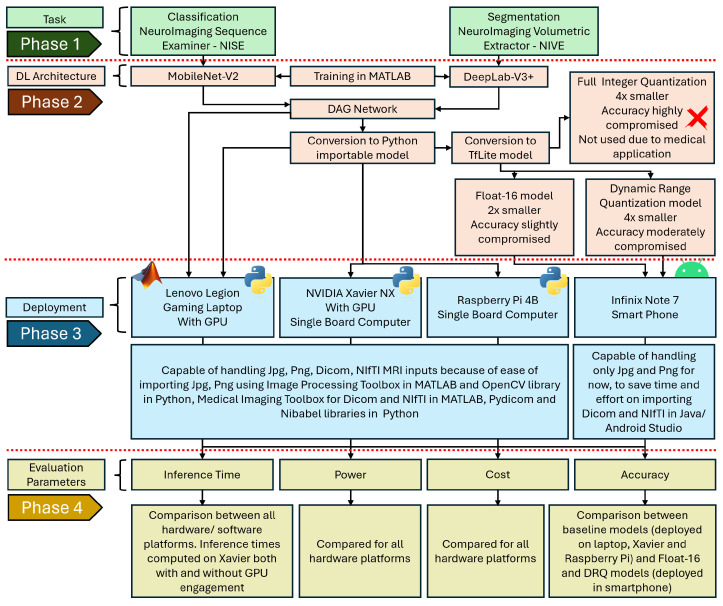
System block diagram outlining the research flow for optimal platform selection in real-time deployment of medical imaging-based CAD tools. The process is divided into four phases: (1) identification and selection of classification and segmentation tasks, (2) selection and training of deep learning architectures (Full Integer Quantization is not used in this research due to the sensitive nature of medical diagnosis applications), (3) integration and deployment of trained DL models onto selected hardware and software platforms after conversion to compatible formats, and (4) evaluation of performance based on established parameters.

The apps are created using MATLAB GUIDE, QT designer/PyQt5, and Android Studio/Java, respectively, for MATLAB, Python and Android. MATLAB and Python are used to run both classification and segmentation apps on Lenovo Legion, and the inference time is recorded. The Python versions of both apps are executed on Raspberry Pi as well as on Xavier with and without using GPU. The results are recorded over 100 iterations. The DRQ TFLite versions of both classification and segmentation models are embedded into Android apps, and the outputs of the DL models are timed in a similar fashion, with the laptop, Xavier and Pi. The overall process is summarized in the block diagram given in Figure 3. The results are presented in the following section.

## 4. Results

The speed of obtaining an inference and segmentation mask in classification and segmentation problems greatly affects the choice of the hardware and software platforms for the real-time deployment of AI systems. Another important and decisive factor is the cost. In this section, the computational speeds are assessed for various platforms ranging from relatively expensive gaming laptops to small and economical handheld gadgets. A cost–benefit analysis is also presented towards the end of this section. In addition, the implications of TFLite conversion of baseline models for the mobile deployment of classification and segmentation applications in terms of accuracy and Dice score is also discussed.

### 4.1. Computational Speed of NISE

**Figure 4:**
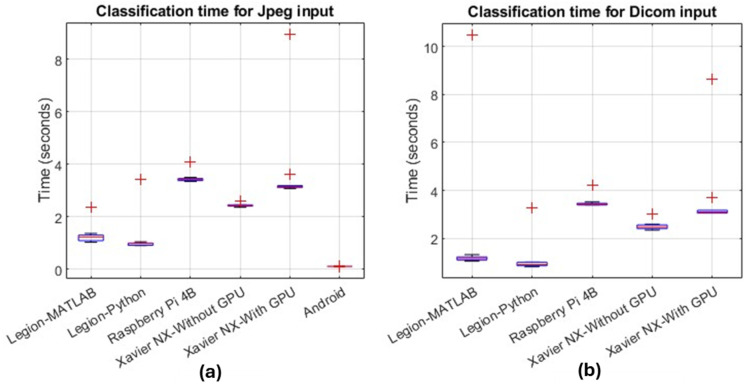
NISE model inference (classification) times using multiple platforms (MATLAB and Python on Lenovo Legion, Python on Raspberry Pi 4B, Python on Xavier NX with and without GPU, and Android) for (**a**) Jpeg and (**b**) Dicom 3-channel MRI inputs with 224 × 224 resolution. The top and bottom of each box represent the upper and lower quartiles, respectively. The red line within the box represents the median value, and the red ‘+’ symbols represent the outliers, resulting from the first execution of the app, which is relatively slower as compared to the subsequent executions.

The classification time of the MRI sequence identification app (NISE) on multiple platforms for Jpeg and Dicom MRI inputs is tabulated in Figure 4. The plots represent the time readings repeated 100 times on each platform. The outliers in each platform result in the first run of the app on the corresponding platform, which is relatively slower than the following executions. This is due to the necessity of performing a set of memory allocations and initializations [13]. The Android app is only programmed to handle Jpeg inputs (not on Dicoms and NIfTIs) since it is sufficient to determine the type of sequence based on a single image.

The performance on Xavier with or without GPU does not vary significantly by changing the wattage and number of cores, and hence only the default mode timing is provided here. Notice how the Android app using the DRQ TFLite version of the model, despite being deployed on a low-specification and low-cost phone, takes the least processing time, closely followed by Xavier. Also, observe that for single-slice computations, the GPU on Xavier does not offer a significant speed boost. The impact of the GPU will be more apparent when dealing with NIfTI MRI whole-brain scans consisting of multiple slices in the segmentation app.

### 4.2. Computational Speed of NIVE

**Figure 5:**
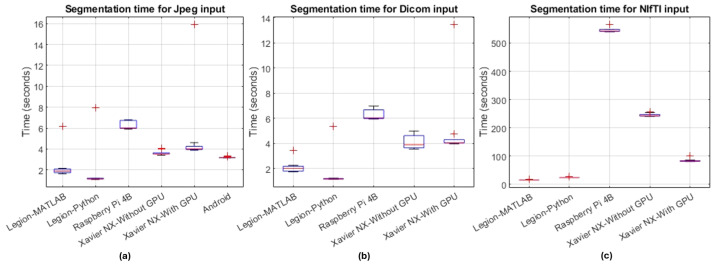
NIVE model segmentation times using multiple platforms (MATLAB and Python on Lenovo Legion, Python on Raspberry Pi 4B, Python on Xavier NX with and without GPU, and Android) for (**a**) Jpeg, (**b**) Dicom and (**c**) NIfTI single channel MRI inputs with 256 × 256 resolution. The top and bottom of each box represent the upper and lower quartiles, respectively. The whiskers extending from the box indicate variability outside the upper and lower quartiles. The red line within the box represents the median value, and the red ‘+’ symbols represent the outliers, resulting from the first execution of the app, which is relatively slower as compared to the subsequent executions.

The computational speed of the segmentation app NIVE on various platforms for Jpeg, Dicom and NIfTI MRI inputs is presented in Figure 5. As in the previous section, data from 100 timed executions of NIVE per platform are depicted here. Image segmentation normally takes longer to produce the result when compared with a classification scenario. The average processing time for NIfTI volumes is higher compared to Jpegs and Dicoms since the whole brain MRI volume used for testing contains 256 slices. Moreover, while handling NIfTI volumes in Python (on laptop, Xavier, and Pi), the MRI slice, brain mask, and skull-stripped images-writing-to-disk times are also included here for all the slices. This writing-images-to-disk part is not implemented in MATLAB, and that justifies its lower processing time than that of Python on Lenovo Legion for NIfTI volume. Notably, the Android smartphone outperforms all devices except the gaming laptop in the segmentation task for Jpeg inputs.

Xavier with GPU outperforms all platforms except the gaming laptop in the segmentation task on NIfTI inputs since the Android phone is not included in this competition. On average, Xavier (with GPU engaged) takes 95 ms per slice for the DL model to infer the segmentation mask, making a total of around 25 s taken for the inference of 256 slices. The remainder of the time is consumed in writing images (256 MRI slices, 256 brain masks, and 256 skull-stripped MRI slices, making a total of 768 Jpeg files) to disk. The impact of GPU on Xavier’s performance is also vivid when processing 256 NIfTI slices since it can be clearly observed to be under 100 s as compared to under 300 s taken by Xavier with GPU disengaged. This speed boost offered by GPU on multiple slices in a NIfTI volume does not impact the processing speed when dealing with single slices in the case of Jpegs and Dicoms, which is evident from the approximately similar processing times with and without the employment of GPU.

### 4.3. Cost and Power Analysis

The prices of the devices used in this research are listed in Table 5 in descending order. As expected, the gaming laptop, despite its modest GPU, generally leads in performance, particularly when used with Python (as shown in Figure 4 and Figure 5). While high-performance machines like this are essential for training complex deep learning models on large-scale medical imaging data, they may be overkill and inefficient if dedicated solely to making inferences from a CAD tool. In such scenarios, edge devices and SBCs offer a more practical solution. However, as seen in Figure 4 and Figure 5, the Raspberry Pi and Xavier (when not utilizing the GPU) exhibit significantly high inference times, ranging from 2.5 s for classifying JPEGs to over 9 min for segmentation tasks with NIfTI volumes. These undesirable delays can be mitigated by using edge devices with GPUs, although this comes with a considerable increase in cost. Nevertheless, this cost remains justifiable compared to gaming laptops, as it is approximately half the price as shown in Table 5.

The performance speed on the other hand, despite almost differing by a multiple of 3, is still comparable i.e., an average of 24.6583 s on Legion and 84.4858 s on Xavier NX with GPU for NIfTI 256 slices as shown in Figure 5. The use of smartphones can further decrease the budget for the deployment of such CAD tools in addition to increasing ease of use. Moreover, no additional hardware or operating system training for medical experts would be required. NVIDIA Xavier and Raspberry Pi on the other hand, despite their compact architecture and low power consumption advantages, are Linux-based systems, which might be an issue for end users not well versed with the operating system. Smart phones come in a wide variety of specifications and prices. The one used in this study is almost the cheapest among all the gadgets used. In addition, the power consumption and operational wattage of such devices is also the least when compared to laptops and SBCs as shown in Table 1. Lenovo Legion consumes 230 W, Xavier a maximum of 20 W, and Infinix Note 7 consumes a meager 18 W, which is comparable to Xavier but around 92% less than that of Legion. Moreover, no dedicated cooling arrangements are required by smartphones as opposed to Legion and Xavier. But unfortunately, all these added benefits come with a catch.

In order to deploy a TensorFlow model on mobile devices, it needs to be converted to a lighter TFLite version. This can be achieved using post-training quantization, which reduces model size and accelerates computations, but on the downside, degrades model’s accuracy. Post-training quantization (used in this research) achieves reduced memory usage and faster speed by quantizing the weights from floating points (32-bit) to lower-precision floating points (16-bit) or integers (8-bit) at conversion time. If the drop in accuracy is too high (generally occurs in smaller networks), workarounds like quantization-aware training can help but at the expense of making additional modifications during model training. A check on accuracy degradation while using TFLite models as opposed to baseline models is therefore mandatory and is given in the following section.

### 4.4. Performance Evaluation of NISE and NIVE—Baseline vs. TFLite

In order to compare the performance of baseline and TFLite models for both classification and segmentation problems, TFLite versions of both models are generated. The size on disk for MobileNetV2 embedded in NISE is significantly reduced from 13 MB (for the baseline model) to 4.9 MB (for float16-TFLite) and further to approximately 3.5 MB (for DRQ-int8-TFLite) after conversion to TFLite. Similarly, for NIVE, the size on disk for DeepLabV3+ decreases from 172 MB (for baseline model) to approximately 84 MB (for float16-TFLite) to around 43 MB (for DRQ-int8-TFLite). This reduction in model size can be extremely helpful for deployment in smartphones due to their limited storage and processing capabilities. Subjects from the IXI [37], MICCAI-21 [35], and ADNI 1.5T [38] datasets are used to evaluate the accuracy of NISE, whereas data from AIH Islamabad [32] are used to test the Dice scores of NIVE baseline and TFLite models.

The precise model file sizes on disk, the details of datasets employed for this task, and the performance results of the baseline and TFLite models for both classification and segmentation tasks are given in Table 6. It is observable from Table 6 that the accuracy for NISE is decreased from 99.84% (using baseline mode) to 99.76% using DRQ-int8 TFLite model. Also notice that the accuracy for float16-TFLite model does not vary from that of the baseline model. This is an important observation since, although the model size is reduced to half as compared to the baseline model, the performance is not altered. For DRQ, on the other hand, a reduction in performance is observable due to further loss in precision.

**Figure 6:**
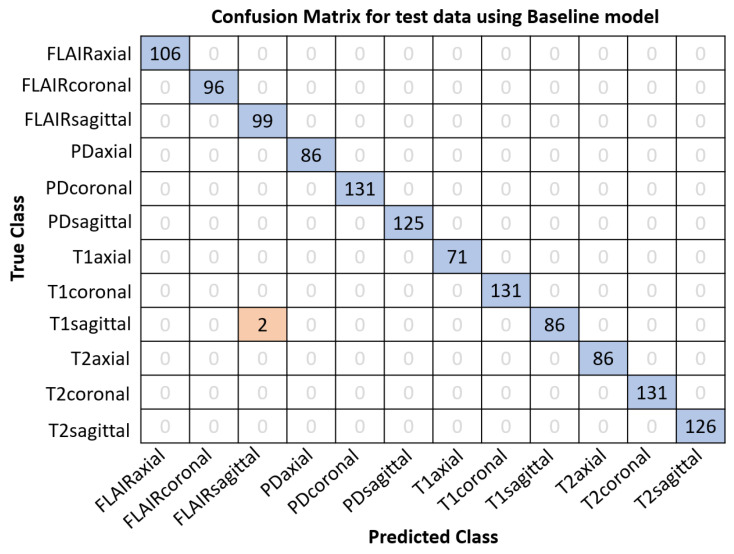
Confusion matrix for NISE baseline classification model on 1276 images. Exactly the same confusion matrix is also seen for the float16 TFLite variant. Notably, only two T1 sagittal MRIs were misclassified as FLAIR sagittal.

**Figure 7:**
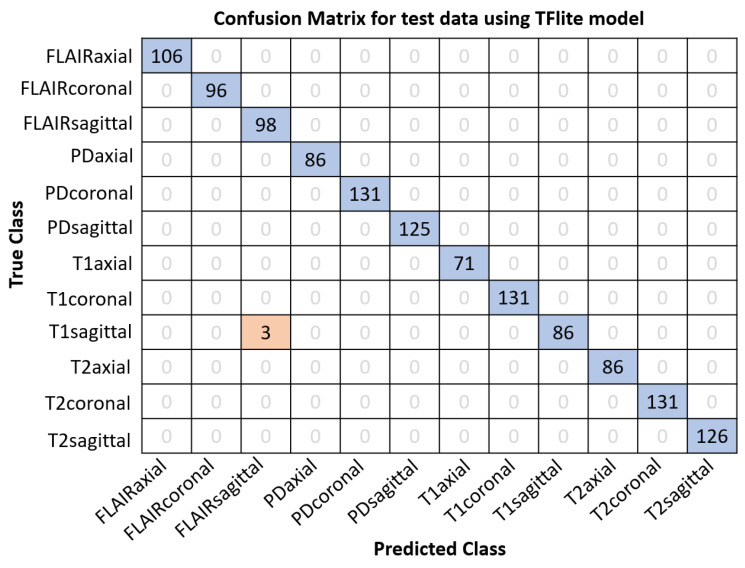
Confusion matrix for NISE DRQ-int8-TFLite classification model on 1276 images. Notably, only three T1 sagittal MRIs were misclassified as FLAIR sagittal.

Similarly, the Dice score for NIVE reduces from 0.9163 (using baseline mode) to 0.9153 using DRQ-int8-TFLite model. The Dice score for float16 TFLite model (0.9162) is observed to be much better than DRQ, as expected. This reduction, despite being unpleasant, is not drastic enough to discard the idea for mobile deployment altogether. To further investigate this degradation in accuracy and Dice, additional tests are conducted. Figure 6 shows the confusion matrix for NISE on 1276 test slices using the baseline model. Notice how two T1-sagittal images are falsely classified as FLAIR-sagittal. The same confusion matrix is achieved for the float16 TFLite model as well. On the other hand, only one additional image out of 1276 is misclassified when using the DRQ-int8-TFLite version of NISE as shown in Figure 7.

**Figure 8:**
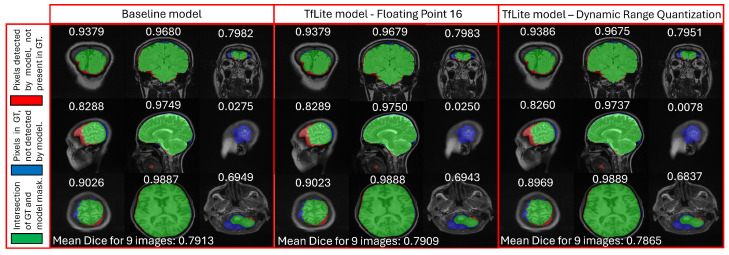
Visualization of segmented brain with its corresponding Dice score of selected slices from 3 subjects of AIH dataset. Comparison of NIVE Dice scores for baseline (**left**), float16 TFLite (**middle**) and DRQ-int8-TFLite (**right**) models. First row contains coronal scans, second row contains sagittal scans, whereas the third row shows axial scans. Green represents the brain region in GT also detected by the model, blue represents the brain in GT not detected by the model, and red represents the brain detected by the model not present in the GT mask.

Similarly, for NIVE, Figure 8 compares the performance of the baseline model and both TFLite variants. This evaluation is conducted using the AIH dataset, which includes three subjects with T1 axial, T2 sagittal, and FLAIR coronal sequences. Out of the 19, 20, and 35 corresponding slices, only three from each category are displayed in Figure 8 (two near-skull slices and one deep-brain slice). The Dice scores for individual slices are shown above each image, with the mean score for this nine-image test cluster found to be 0.7913 for the baseline model and 0.7865 for the corresponding DRQ-int8-TFLite variant. The float16 TFLite model demonstrates intermediate performance, with a Dice score of 0.7909. In Figure 8, the green color indicates brain regions correctly detected by the models, blue represents brain regions present in the GT but missed by the models, and red shows brain regions detected by the models but absent in the GT. The baseline model and both TFLite variants exhibit very similar performance, with only minor differences in Dice scores (with the TFLite models slightly underperforming the baseline in most cases).

The marginal reduction in performance observed in the TFLite variants of the baseline classification and segmentation models, upon closer examination, appears to be reasonably acceptable. However, considering the high sensitivity of the application in question—namely CAD, where human lives may be at stake—it is reassuring to know that techniques like quantization-aware training exist. These methods allow us to avoid even the slightest compromise on performance and accuracy while still enabling the deployment of such CAD tools on mobile devices.

In addition to evaluating the classification and segmentation accuracy of the deployed models, the inference time is also recorded as shown in Table 5. Notably, the average classification inference time for JPEG inputs on the Infinix Note 7 (using DRQ TFLite version of the baseline model) is found to be 0.1068 s, which is faster than that on the Lenovo Legion (using the baseline model). However, for segmentation, the time taken by the Android device is longer than that of the GPU-equipped laptop, though still faster than single-slice processing on SBCs, both with and without using GPUs.

Moreover, the deep learning models embedded within mobile apps can also be served over the cloud using platforms like Google Firebase [40]. This approach facilitates the deployment of updated models to CAD tool users in real-time, without requiring a full app reinstallation. As a result, smartphones emerge as an excellent platform for the real-time deployment of medical imaging-based CAD tools, offering an optimal balance between cost, flexibility, and performance, closely followed by GPU-equipped SBCs.

## 5. Conclusions

The advancement of mobile and edge computing devices has significantly enhanced their ability to host complex deep learning models trained on extensive datasets, enabling real-time, accurate inferences. This study evaluated several such devices as platforms for deploying medical imaging-based CAD tools in real-time hospital operations. The devices were tested for both classification and segmentation tasks using brain structural MRI data in various file formats. Among them, smartphones emerged as an optimal platform due to their low cost, ease of use, reasonable operational speed, and satisfactory accuracy. Additionally, SBCs equipped with GPUs demonstrated promising performance, though with minor trade-offs in cost, power consumption, and usability. Deploying CAD tools on these devices can provide crucial support to medical professionals in underdeveloped and remote areas with limited resources and internet access, thereby enabling rapid and accurate differential diagnoses that can save lives.

## Figures and Tables

**Table 1:** Specifications of the hardware platforms for the deployment of NeuroImaging Sequence Examiner (NISE) and NeuroImaging Volumetric Extractor (NIVE).

Hardware	Processor	GPU	RAM	OS	Storage	Power
Lenovo Legion	Intel(R) Core (TM) i7- 9750H CPU @ 2.60 GHz	NVIDIA GeForce GTX 1660 Ti	16 GB	Microsoft Windows 11 Home	512 GB SSD and 1 TB SATA	230 W
NVIDIA Jetson Xavier NX	6-core NVIDIA Carmel ARM®v8.2 64-bit CPU	384- core NVIDIA Volta™ GPU with 48 Tensor Cores	16 GB	Jetpack 5.1.2 Ubuntu 20.04.6 (Focal Fossa)	16 GB eMMC 5.1 internal storage and 128 GB NVME SSD	10 W/15 W/20 W
Raspberry Pi 4B	Broadcom BCM2711, quad-core Cortex-A72 (ARM v8) 64-bit SoC @ 1.5 GHz processor	-	8 GB	Debian GNU/Linux 12 (Bookworm)	32 GB MicroSD card	15 W
Infinix Note 7	Octa-core (2x2.0 GHz Cortex-A75 6x1.7 GHz Cortex-A55) CPU	Mali-G52 2EEMC2 GPU	6 GB	Android 10	128 GB internal storage	18 W

**Table 2:** Software and libraries on different hardware platforms.

Hardware	Software	Version
Lenovo Legion	OS	MS Windows 11 Home
	MATLAB	R2022b
	Python	3.10.7
	Android Studio	Giraffe 2022.3.1
	TensorFlow	2.10.0
	TensorFlow-GPU	2.10.0
	NVIDIA CUDA	11.2
	cuDNN	8.1
Xavier NX	Jetpack	5.1.2
	Ubuntu	20.04.6 (Focal Fossa)
	NVIDIA SDK Manager	2.0.0
	NVIDIA CUDA	11.4
	cuDNN	8.6
	Python	3.8.10
	TensorFlow	2.12.0
Raspberry Pi 4B	Debian GNU/Linux	12 (Bookworm)
	Python	3.11.2
	TensorFlow	2.15.0
Infinix Note 7	OS	Android 10
Supporting Python libraries: PyQT5, pydicom, tkinter, nibabel, openCV		

**Table 3:** Datasets used for the training, testing, and validation of NIVE segmentation app (top), NISE classification app (middle), and for Inference Time Computation on NIVE and NISE (bottom).

**NIVE (Segmentation)**											
	**Type of MRI**					**Mask**		**No. of Images or Patients**		**Utilization for NIVE**	
**Dataset**	**FLAIR**	**T1-W**	**T2-W**	**PD**	**Condition**	**Lesion**	**Brain**		**No. of Slices**	**Training + Validation**	**Testing**
NFBS [33]	-	√	-	-	Multiple	-	√	125 volumetric data	6000	√	√
SynthStrip [31]	√	√	√	√	Not specified	-	√	582 volumetric data	118,606	√	√
MICCAI 2016 [34]	√	√	√	-	Multiple Sclerosis	√	√	53-patients	34,082	√	√
Baghdad [36]	√	√	√	-	Multiple Sclerosis	√	-	60-patients	12 / sequence	-	√
AIH Islamabad [32]	√	√	√	-	Multiple	-	√	3-patients	Varies	-	√
**NISE (Classification)**											
**Dataset**	**Type of MRI**				**Condition**	**Orientation**			**Utilization for MRISI**		
	**FLAIR**	**T1-W**	**T2-W**	**PD**		**Axial**	**Coronal**	**Sagittal**	**Training + Validation**	**Testing**	
NFBS	-	6000	-	-	Multiple	2000	2000	2000	√	√	
SynthStrip	389	52,017	30,789	28,681	Not specified	38,510	40,071	33,295	√	√	
MICCAI 2016	34,083	-	-	-	Multiple Sclerosis	11,302	15,205	7576	√	√	
IXI [37]	-	-	343	342	Multiple	172	262	251	-	√	
MICCAI 2021 [35]	303	-	-	-	Multiple Sclerosis	106	96	101	-	√	
ADNI 1.5 Tesla [38]	-	288	-	-	Alzheimer’s	71	131	86	-	√	
AIH Islamabad	35	19	20	-	Multiple	19	35	20	-	√	
**Inference Time Computation on NIVE and NISE**											
**Dataset**	**Type of MRI**				**Condition**	**Orientation**			**Utilization for NIVE and NISE**		
	**FLAIR**	**T1-W**	**T2-W**	**PD**		**Axial**	**Coronal**	**Sagittal**	**Time Computation**		
SynthStrip	-	81	-	-	Not specified	81	-	-	√		
AIH Islamabad	-	19	-	-	HIV	19	-	-	√		

**Table 4:** TFLite optimizations: post-training quantization options and quantization-aware training methods.

Technique	Data Requirements	Size Reduction	Accuracy	Supported Hardware
Post-training Float16 Quantization (F16Q)	No data	Up to 50%	Insignificant accuracy loss	CPU, GPU
Post-training Dynamic Range Quantization (DRQ)	No data	Up to 75%	Smallest accuracy loss	CPU, GPU (Android)
Post-training Full Integer Quantization (FIQ)	Unlabeled representative sample	Up to 75%	Small accuracy loss	CPU, GPU (Android), EdgeTPU, Hexagon DSP
Quantization-aware training (QAT)	Labeled training data	Up to 75%	Smallest accuracy loss	CPU, GPU (Android), EdgeTPU, Hexagon DSP

**Table 5:** Make, model, and cost of devices used, along with the mean inference time for classification and segmentation tasks on Jpeg inputs. Listed prices (in Malaysia Ringgit) are rounded to the nearest hundreds.

Device	Model	Cost (MYR)	Mean Inference Time (in sec)	
			Classification	Segmentation
Laptop	Lenovo Legion Y545	7000	1.2074	1.8241
SBC	NVIDIA Jetson Xavier NX 16 GB	4000	3.7627	5.2641
Phone	Infinix Note 7	500	0.1068	3.2023
SBC	Raspberry Pi 4B	300	3.4747	6.2162

**Table 6:** Comparison between baseline and TFLite models for NISE and NIVE in terms of file size, accuracy, and Dice score.

Task	Architecture	Testing Dataset	Number of Test Images	DL Model	Size on Disk	Accuracy/Dice
Classification (NISE)	MobileNetV2	IXI, MSSEG-2, ADNI 1.5 T	1276 images belonging to 12 classes	Baseline	13,472 KB	99.84%
				TFLite-F16Q	4996 KB	99.84%
				TFLite-DRQ	3647 KB	99.76%
Segmentation (NIVE)	DeepLabV3+	AIH Islamabad Dataset	74 images (T1 Axial 19, T2 Sagittal 20, FLAIR Coronal 35)	Baseline	176,857 KB	0.9163
				TFLite-F16Q	86,241 KB	0.9162
				TFLite-DRQ	43,625 KB	0.9153

## Data Availability

The original contributions presented in the study are included in the article; further inquiries can be directed to the corresponding author.

## Abbreviations

The following abbreviations are used in this manuscript:

Advanced International Hospital	AIH
Artificial Intelligence	AI
Computed Tomography	CT
Computer-Aided Diagnosis	CAD
Convolutional Neural Network	CNN
Deep Learning	DL
Deep Neural Network	DNN
Dice Similarity Coefficient	DSC
Directed Acyclic Graph	DAG
Dynamic Range Quantization	DRQ
False Negative	FN
False Positive	FP
Fluid-Attenuated Inversion Recovery	FLAIR
Graphical User Interface Development Environment	GUIDE
Graphics Processing Unit	GPU
Ground Truth	GT
Machine Learning	ML
Magnetic Resonance Imagining	MRI
Multiple Sclerosis	MS
NeuroImaging Sequence Examiner	NISE
NeuroImaging Volumetric Extractor	NIVE
Neuromyelitis Optica	NMO
Positron Emission Tomography	PET
Post-training Quantization	PTQ
Proton Density	PD
Quantization-Aware Training	QAT
Single-Board Computer	SBC
Tensor Processing Unit	TPU
True Negative	TN
True Positive	TP
